# Potential Effects of High Temperature and Heat Wave on *Nanorana pleskei* Based on Transcriptomic Analysis

**DOI:** 10.3390/cimb45040192

**Published:** 2023-04-03

**Authors:** Tao Zhang, Zhiyi Niu, Jie He, Peng Pu, Fei Meng, Lu Xi, Xiaolong Tang, Li Ding, Miaojun Ma, Qiang Chen

**Affiliations:** 1 Department of Animal and Biomedical Sciences, School of Life Science, Lanzhou University, Lanzhou 730000, China; 2 Department of Animal Science, School of Life Sciences and Engineering, Southwest University of Science and Technology, Mianyang 621010, China; 3 State Key Laboratory of Grassland and Agro-Ecosystems, School of Life Sciences, Lanzhou University, Lanzhou 730000, China

**Keywords:** the Qinghai-Tibet plateau, physiological effects, global warming, hypometabolic states, energy supply

## Abstract

**Simple Summary:**

Understanding how native amphibians of the Qinghai-Tibet plateau respond to pressures and their copying mechanisms could be essential for predicting their destiny in the face of climate change. A liver transcriptome was constructed to find those coping mechanisms of *Nanorana pleskei*. Frogs switched into hypometabolic states under high-temperature stress. However, the energy supply was basically normal to sustain the highly energy-demanding metabolic functions during heat waves. Genes were more transcriptionally suppressed for combating these long-term adverse environments to survive. High temperatures are more harmful to frogs than heat waves.

**Abstract:**

In the context of climate change, understanding how indigenous amphibians of the Qinghai-Tibet plateau react to stresses and their coping mechanisms could be crucial for predicting their fate and successful conservation. A liver transcriptome for *Nanorana pleskei* was constructed using high-throughput RNA sequencing, and its gene expression was compared with frogs acclimated under either room temperature or high temperature and also heat wave exposed ones. A total of 126,465 unigenes were produced, with 66,924 (52.92%) of them being annotated. Up to 694 genes were found to be differently regulated as a result of abnormal temperature acclimatization. Notably, genes belonging to the heat shock protein (*HSP*) family were down-regulated in both treated groups. Long-term exposure to high-temperature stress may impair the metabolic rate of the frog and trigger the body to maintain a hypometabolic state in an effort to survive challenging times. During heat waves, unlike the high-temperature group, mitochondrial function was not impaired, and the energy supply was largely normal to support the highly energy-consuming metabolic processes. Genes were more transcriptionally suppressed when treated with high temperatures than heat waves, and the body stayed in low-energy states for combating these long-term adverse environments to survive. It might be strategic to preserve initiation to executive protein activity under heat wave stress. Under both stress conditions, compromising the protection of *HSP* and sluggish steroid activity occurred in frogs. Frogs were more affected by high temperatures than by heat waves.

## 1. Introduction

Amphibians are considered as an excellent study group to further improve our understanding of genomics, because they exhibit a wide range of reproductive strategies [1,2], occupy the majority of ecoregions on earth, have a highly diverse range of morphology, and are the most endangered group of vertebrates globally [3]. Climate change can be more harmful to amphibians than other groups of vertebrates and is one of the major causes of the extinction of ectotherms [4]. A growing number of studies have proved that the global climate is changing rapidly, with unexpected implications [5]. It has been found that climate change has a wide range of effects on natural systems [6]. Influences on abundance, distribution [7,8,9,10], and morphology [11,12] have been recorded at the species level. Climate change may lead to a major shift in spatial patterns of Chinese amphibian diversity [13]. Changes in biological features and genetic diversity are also possible [14]. Temperature rise is the most important sign of climate change; one of its consequences is the occurrence of extreme phenomena such as heat waves [15,16].

Next-generation sequencing (NGS) has grown more affordable, allowing researchers to study the transcriptome of any organism [17,18]. The use of NGS in amphibians allows researchers to make crucial findings and discoveries at the molecular level of plasticity, as well as the expansion of sequencing resources [19,20]. RNA-sequencing was used to investigate the molecular basis of phenotypic plasticity in spotted seatrout *Cynoscion nebulosus* populations exposed to acute cold and heat stress [21]. Comparative transcriptome analysis of three acclimatized groups of *Quasipaa boulengeri* tadpoles found key differentially expressed genes which coincided with phenotype and hormone level and offered a suitable rearing temperature [22]. Transcriptomic profiles and protein-protein interaction analysis of heat-treated *Buergeria otai* tadpoles exhibit global suppression of DNA transcription and mRNA translation that ensure them to survive high temperatures [23]. Similarly, through transcriptome analysis, it was found that genes involved in secretion and defensive function are highly expressed in the skin of *Rana chensinensis* [24]. Sun et al. found some important pathways responding to heat stress in the liver tissues of rainbow trout *Oncorhynchus mykiss* [25]. *Nanorana pleskei* is one of the most significant apex predators in wetland ecosystems, playing crucial functional roles in community structuring and ecological equilibrium [26,27]. According to our fieldwork, there are three amphibian species, *Bufo gargarizans*, *Rana kukunoris*, and *N. pleskei* in Awancang marsh. *Nanorana pleskei* is classified as near threatened (NT) in ICUN red list. It is not listed in the national list for major protective wild animals of China, however, the frog is a species of the collection of “three animals” which means terrestrial wildlife with important ecological, scientific and social values. Thus, it is also protected by the Wildlife Protection Law of the People’s Republic of China. However, the distribution range of *N. pleskei* is the smallest among the three speices (https://www.amphibiachina.org (accessed on 28 March 2023)), and this species could be more likely affected by the climate change.

*Nanorana pleskei* (Dicroglossidae) is an indigenous species of the Qinghai-Tibet plateau, living among puddles and ponds in swampy environments ranging from 3300–4500 m above sea level [28]. During the day, adult frogs lurk near streams or under grasses in swampy places; at night, they crouch along water edge or amid grasses, feeding on tiny insects and their larvae [27,29]. At present, other research has been conducted on the ecology and physiology of this species. Their main spawning strategies include shallow waters, decentralized spawning patterns, and egg attachment to an appropriate distance to the water surface. Therefore, drought caused by rising temperatures and fluctuating precipitation may induce *N. pleskei* numbers to decline [30]. Remarkable numbers of convergent and parallel amino acid substitutions of the *MYBPC2* gene exist in *Bufo gargarizans*, *R. kukunoris,* and *N. pleskei* [31]. Cold acclimation reduces antioxidant defense in *N. pleskei* [32]. However, *N. pleskei* may have well adapted to the significant temperature fluctuation, according to a study on the impact of high temperatures and heat waves on thermal biology, locomotor performance, and antioxidant system [33]. The range of tolerated temperature, thermal preference, and critical thermal maximum all dramatically increased after two weeks of heat wave acclimation, whereas the critical thermal minimum significantly dropped [33].

Heat shock protein (*HSP*) production is a typical event in prokaryotic and eukaryotic cells under stress conditions such as increased temperature and oxidative stress [34]. *HSP* genes were discovered to be downregulated in Oklahoma salamander *Eurycea tynerensis* [35] and Ota’s stream tree frog *B. otai* [23] under heat stress.

The study of transcriptomics in no-model animals gained momentum in the past decade. However, no gene-level research was conducted on *N. pleskei* in regard to temperature adaptation under the background of climate change. Hence, in this study, we try to determine the regulation of gene expression in *N. pleskei* under high temperatures and heat waves using comparative transcriptomics. What physiological impacts and copying mechanisms are there in the presence of a hostile environment? Answering the above issues at the transcription level may deepen our understanding of amphibian physiology and provide insight into functional changes of *N. pleskei* when facing an increased temperature or heat wave.

## 2. Materials and Methods

### 2.1. Animal Acclimation

**Figure 1:**
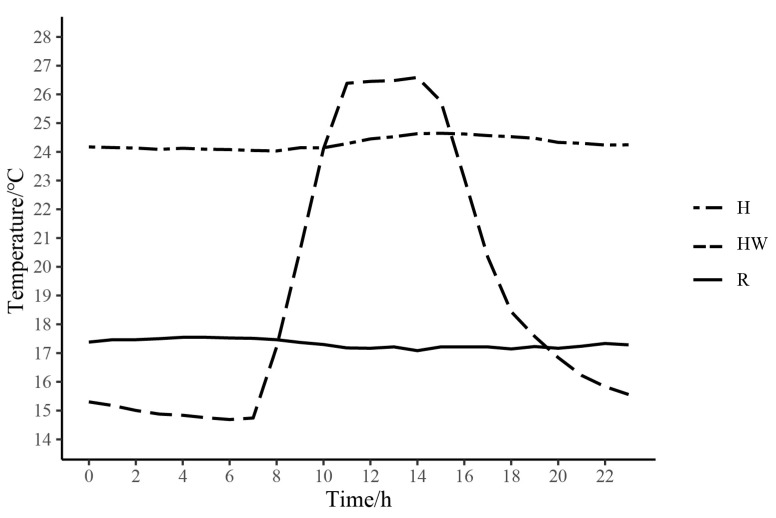
Changes in mean temperature of 24 h in the room temperature (R), high temperature (H), and heat wave (HW) groups for two weeks. Real-time temperatures were recorded by three iButtons (DS1921, MAXIM Integrated Products Ltd., USA) which were fixed in the middle of each box.

**Figure 2:**
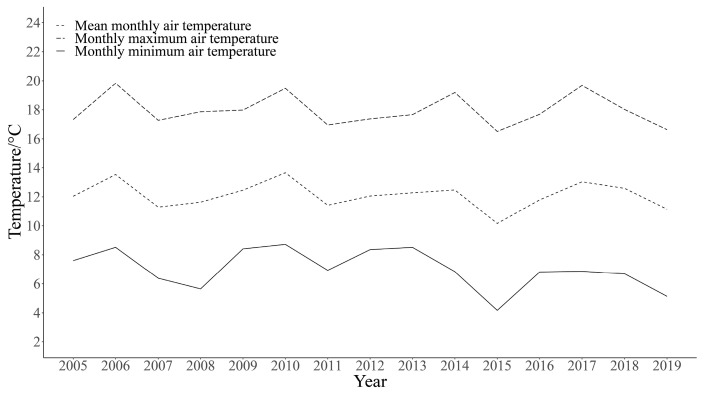
Monthly maximum air temperature, mean monthly air temperature, and monthly minimum air temperature in July in Maqu County from 2005 to 2019.

Eighteen male frogs were collected from Awancang (33.795° N, 101.76° E, 3501 m asl), Maqu County, Gansu Province, China, in July 2020. Frogs were transported to the laboratory at Gannan Grassland Ecosystem National Observation and Research Station, which is near (15 km) the sampling site. Snout-to-vent length (SVL, 3.09 ± 0.73 cm) and body mass (BM, 3.61 ± 0.30 g) of all the frogs were measured. Frogs were randomly assigned into three acclimating groups (six frogs in each group), room temperature (R, 17 °C) group, high temperature (H, 25 °C) group and heat wave (HW, maximum (27 °C) and minimum (14 °C) group (Figure 1). Frogs of each group were kept in one plastic container (46 × 34 × 26 cm, with a platform and water). The containers of both high temperature and heat wave groups were put in two separate wooden cabins (62.5 × 47.8 × 40.1 cm) equipped with automatic temperature control systems (AI-719P, Xiamen Yudian Automation Technology Co., Ltd., Shenzhen, China). The temperature inside the boxes was monitored by iButtons (DS1921, MAXIM Integrated Products Ltd., San Jose, CA, USA). In order to simulate the temperature variation experienced by frogs during the active period, the heat wave group was defined on the basis of the monthly maximum (17.9 ± 1.1 °C) and minimum (7.0 ± 1.4 °C) average temperatures in July in Maqu County from 2005 to 2019 (Figure 2). With the automatic temperature control system, we set the temperature specific to the time in the heat wave group. It starts to heat at 7:30 every day. The rate of heating was controlled automatically. The set value for 11:00 to 15:00 was 27 °C.

Water used for the frogs was obtained from a nearby stream. Insects such as mosquitoes, flies, and locust larvae were collected from grassland to feed the frogs. The water was changed every three days after the frogs were fed. Meantime, the leftover insects were cleared. The photoperiod is 12 L: 12 D. Three male frogs per group were randomly selected as representatives after two weeks of acclimation (16–30 July 2020) for analysis. The sampling timepoint for the heat wave group was 7:20 to 7:30, and the temperature was not evaluated in the box by an automatic temperature control system. Frogs from each group were sacrificed by quick decapitation without anesthesia [36]. Then, the livers were collected and immediately frozen in liquid nitrogen. The room temperature group was used as the control group.

Liver samples were transported to Novogene (Beijing, China) with a dry ice box. The library preparations were sequenced on an Illumina Hiseq platform, and 150 paired-end reads were generated. Clean data were obtained from raw data by removing reads containing adapter, reads containing ploy-N, and low-quality reads. All the subsequent analyses were conducted using clean, high-quality data.

### 2.2. Assembly and Evaluation

Transcriptome assembly was performed using Trinity (Brian J Haas, version v2.11.0, Cambridge, MA, USA) [37,38] with these parameters (min_kmer_cov 3 min_glue 4 max_chrysalis_cluster_size 31 min_contig_length 300 bfly_opts ‘-V 5 edge-thr = 0.1 stderr’) and all other parameters were set to default. Next, the reference transcript was produced by eliminating redundant contigs by CD-HIT [39]. Transrate [40] was used to obtain the average transcript length and N50 values of the de novo transcriptome assemblies.

### 2.3. Function Annotation

Due to the lack of genomic resources of *N. pleskei*, we combined protein data from five species to create a reference dataset for gene annotation, which includes *N. parkeri*, *R. temporaria*, *Leptobrachium leishanense*, *Xenopus tropicalis*, and *X. laevis*. The complete *X. laevis* and *X. tropicalis* proteomes were downloaded from the Uniprot database [41]. The protein data and annotation files of *N. parkeri* and *L. leishanense* were downloaded from figshare (https://figshare.com/projects/Genomic_data_of_Nanorana_parkeri/116061 (accessed on 13 August 2021); https://figshare.com/articles/dataset/Genome_assembly_of_Leptobrachium_leishanense/8019986 (accessed on 10 August 2020)), and protein data of *R. temporaria* was downloaded from NCBI (National Center for Biotechnology Information). Transcripts were annotated to the reference dataset by BLAST [42] similarity using blastx, using the maximum value for identity and minimum value for E-value, and with the option “-subject_besthit -max_target_seqs 1 -evalue 1e-5” enabled to keep just the best match for the best alignment of each query sequence.

### 2.4. Analysis of Differentially Expressed Genes and Functional Enrichment

The expression level of the transcripts was quantified by mapping the Illumina reads to the reference sequences by RSEM software (Bo Li, version 1.3.3, Madison, WI, USA) with the bowtie2 parameter and other default parameters [43]. The differential gene expression analysis was conducted using DESeq2 package (Michael I Love, version 1.30.0, MA, USA) [44]. Differentially expressed genes (DEGs) with the |log2(fold change)| ≥ 1 and adjust *p*-value ≤ 0.05 were considered significant. GO term and KEGG pathway enrichment analysis was performed in the clusterProfiler package (Guangchuang Yu, version 4.4.2, Guangzhou, China) in R [45,46].

## 3. Results

### 3.1. De Novo Assembly and Annotation

A total of 192,692,377 raw reads, which range from 18,154,470 to 23,538,026, were generated for nine *N. pleskei* samples. The RIN values were all greater than 7.1, with the exception of one sample, which yielded near the amount of data as the other samples. After filtering, 186,540,630 reads which range from 17,433,117 to 22,782,142 were retained. The percentage of base with greater than Q30 (phred quality score; Q score) in each sample retained were all above 89.06 (Table S1). Finally, a total of 126,465 unigenes (total length of 149,653,736 bp) were generated with a mean length of 1183 bp and an N50 length of 1951 bp.

By combining protein data from five different species, we were able to create a reference dataset that represented the main lineages of amphibian species, which updated the genome assembly using the latest sequencing methods. After the cut-off for identity and E-value, 126,465 transcripts of *N. pleskei* were annotated to 66,924 genes. A total of 33,668 annotated transcripts had an E-value below 1 × 10^−50^.

### 3.2. Differential Gene Expression and Pathway Enrichment

**Figure 3:**
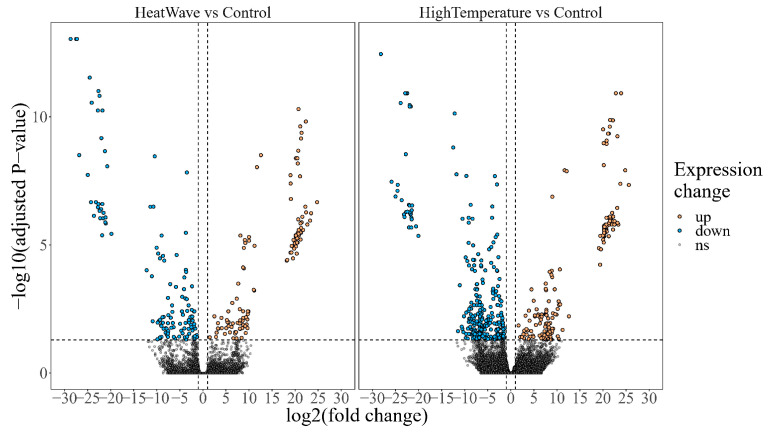
Volcano plot depicting transcriptomic change in response to a heat wave and high temperature, respectively. Horizontal dotted line represents the adjusted *p*-value = 0.05, and vertical dotted lines represent the absolute value of log2(fold change) = 1.

Read mapping ratios of nine sequence data ranged from 87.13% to 91.16%. Compared with the control group, 453 DEGs (290 down, 163 up) and 241 DEGs (122 down, 119 up) were identified in the H group and HW group, respectively (Figure 3).

**Figure 4:**
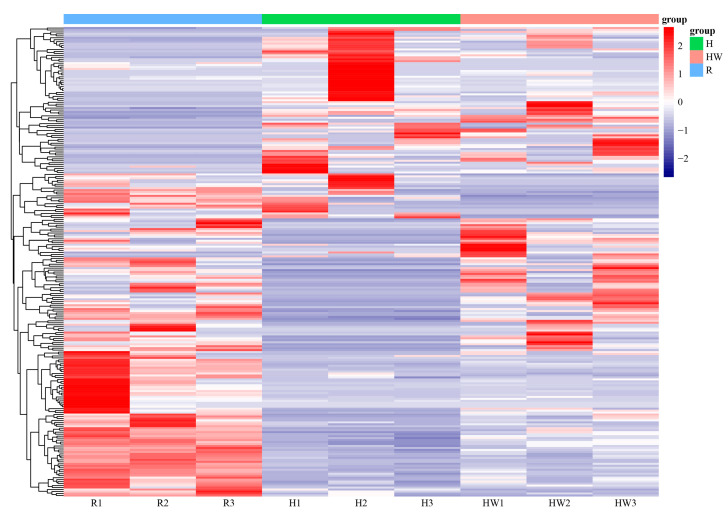
Heat map of different genes with KEGG annotation in different samples. R: room temperature, H: high temperature, HW: heat wave.

However, the number of DEGs with annotation information was reduced to 240 (Figure 4), which are included in enzymes, membrane trafficking, ubiquitin system, messenger RNA biogenesis, transcription factors, mitochondrial biogenesis, lipid biosynthesis proteins, and amino acid-related enzymes.

**Figure 5:**
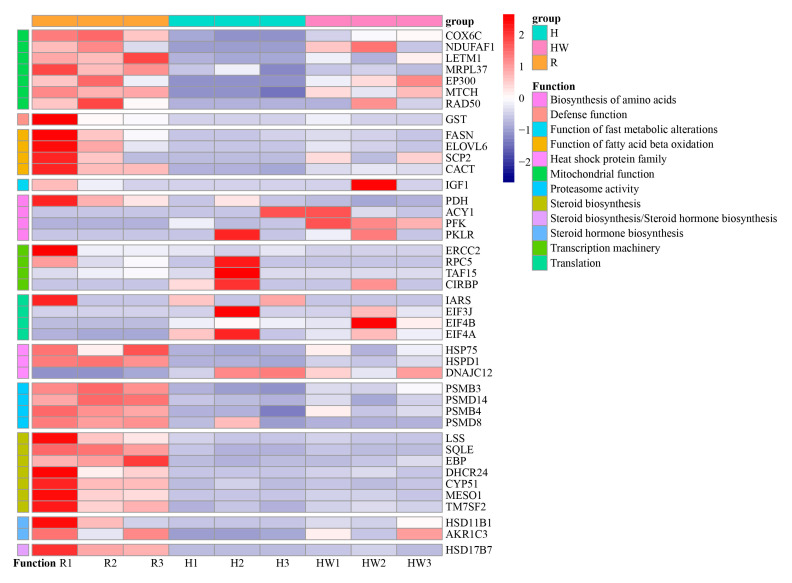
Heat map of different genes with detailed function in different samples. R: room temperature, H: high temperature, HW: heat wave.

In response to high-temperature stress, the expression of cytochrome c oxidase sub-unit 6c (*COX6C*), NADH dehydrogenase [ubiquinone] 1 alpha subcomplex assembly factor 1 (*NDUFAF1*) and EF-hand domain-containing protein 1(*LETM1*), elongation factor Ts (*tsf*), large subunit ribosomal protein L37 (*MRPL37*), E1A/CREB-binding protein (*EP300*) and mitochondrial carrier MTCH, huntingtin (*HD*) in mitochondrial biogenesis were downregulated. Meanwhile, the DNA repair protein RAD50 (*RAD50*) gene and Glutathione S-transferase (*GST*) were downloaded in the H group (Figure 5, Table S4). In addition, fatty acid synthase (*FASN*) and elongation of very long chain fatty acids protein 6 (*ELOVL6*) were downregulated. Meanwhile, sterol carrier protein 2 (*SCP2*), which is responsible for the function of fatty acid beta-oxidation, was down-regulated.

Under heat wave, the expression of the pyruvate dehydrogenase (*PDH*) gene was lowered. Similarly, aminoacylase (*ACY1*), 6-phosphofructokinase 1 (PFK), and pyruvate kinase isozymes R/L (*PKLR*) in the biosynthesis of amino acids pathway were upregulated. DNA excision repair protein ERCC-2 (*ERCC2*), DNA-directed RNA polymerase III subunit RPC5 (*RPC5*), and transcription initiation factor TFIID subunit 15 (*TAF15*) in transcription machinery were downregulated. Translation initiation factor 3 subunit J (*EIF3J*), translation initiation factor 4B (*EIF4B*), and translation initiation factor 4A (*EIF4A*), which belong to translation factors, were upregulated. DNA excision repair protein ERCC-2 (*ERCC2*), DNA-directed RNA polymerase III subunit RPC5 (*RPC5*), and transcription initiation factor TFIID subunit 15 (*TAF15*) in transcription machinery were downregulated. *HSP75*, *HSPD1*, 20S proteasome subunit beta 3 (*PSMB3*), 26S proteasome regulatory subunit N11 (*PSMD14*), 20S proteasome subunit beta 7 (*PSMB4*), and 26S proteasome regulatory subunit N12 (*PSMD8*) in proteasome were also downregulated in these acclimated groups.

Moreover, under both accumulated groups, lanosterol synthase (*LSS*), squalene monooxygenase (*SQLE*), 17beta-estradiol 17-dehydrogenase/3beta-hydroxysteroid 3-dehydrogenase (*HSD17B7*), cholestenol Del-ta-isomerase (*EBP*), Delta24-sterol reductase (*DHCR24*), sterol 14alpha-demethylase (*CYP51*), methylsterol monooxygenase (*MESO1*), and Delta14-sterol reductase (*TM7SF2*) in steroid biosynthesis (ko00100) were significantly downregulated. Corticosteroid 11-beta-dehydrogenase isozyme 1 (*HSD11B1*), 17beta-estradiol 17-dehydrogenase/3beta-hydroxysteroid 3-dehydrogenase (*HSD17B7*), and aldo-keto reductase family 1 member C3 (*AKR1C3*) in steroid hormone biosynthesis (ko00140) were also notably downregulated.

No significantly enriched KEGG pathways were retained according to the corrected *p*-value, with the exception of steroid biosynthesis (ko00100), which was shown to be over-represented by down-regulated DEGs of H and HW groups (Table S2). Despite the modest number of meaningful findings, some clues may be found from the GO results (Table S3). The most overrepresented pathways are the glutamine metabolic process, mRNA transport, RNA binding, lipid biosynthetic process, carbohydrate metabolic process, mitochondrial biogenesis, and regulation of translation.

## 4. Discussion

Temperature is one of the most pervasive factors influencing the physiology of ectotherms. Ectothermic animals undergo molecular, cellular, and physiological adjustments that ensure functional integrity during abnormal temperature exposure [47,48]. Thermal plasticity (both active and passive) can result from changes across levels of the biological hierarchy, from the molecular level. The molecular processes involved in hepatic responses to extreme temperature, particularly heat waves, are complicated and poorly understood molecular processes in *N. pleskei*. In this study, we acclimated *N. pleskei* with constant high temperature and heat waves and measured their expression profiles via transcriptome sequencing.

### 4.1. Treatment-Specific Genes and KEGG Pathways

Impairment of mitochondrial function may result in lower energy availability [49,50]. *GST* is an enzyme that utilizes glutathione (*GSH*) to play an important role in defense mechanisms [51]. *GST* can be overexpressed to provide resistance under intracellularly induced oxidative stress [52]. However, in the present study, a sensitive downregulated response of *GST* was observed in the livers of frogs exposed to high temperatures for two weeks, which might indicate that this enzyme would not work in defense mechanisms. Coincidently, *GST* showed no change in *R. chensinensis* exposed to trichlorfon [53]. Genes associated with fatty acid synthase were dysfunctional. We speculated that fatty acid did not act as an energy storage material and had no excess energy to synthesize in high-temperature stress. The acylcarnitine transporter (*CACT*), which is responsible for the function of the carnitine-acyl carnitine antiporter, was down-regulated. Meanwhile, no DEG was found in fatty acid degradation, which supplies large quantities of acetyl-CoA that could be in agreement with impaired mitochondrial function. The marked deposition of triacylglycerol (TAG, TG) as a fuel source in the liver triglycerides of *N. parkeri* in the summer implies that lipids are used to prepare for overwintering [54]. Conversely, inactive lipid metabolism at high temperatures may benefit the frogs in unfavorable conditions to avoid cell damage caused by lipid peroxidation in the body [55,56]. In the H group, insulin-like growth factor 1 (*IGF1*), which plays a critical role in producing fast metabolic alterations and is mostly produced by the liver [57], was lowered. The prolonged heat stress may impair the metabolic response of the body and trigger the body to keep a low metabolic state in the hope of surviving challenging times.

Under heat waves, *PDH* regulates the entry of Acetyl-CoA units into the TCA cycle and gluconeogenesis [58]. Similarly, three key genes in the biosynthesis of amino acids pathway were upregulated. As a result, heat wave stress may lead pyruvate flow to amino acids as material. The transcribing machinery was not operational. However, cold-inducible RNA-binding protein (*CIRBP*) was increased in the HW group, helping cells stabilize mRNAs and facilitate translation [59]. Aminoacyl-tRNAs are translation substrates and are critical in determining how the genetic code is translated into amino acids [60]. Aminoacyl-tRNA synthetases in aminoacyl-tRNA biosynthesis may not be affected except isoleucyl-tRNA synthetase (*IARS*), and three transcription initiation factors had higher expression. Similarly, eukaryotic initiation factor-2 (*eIF2*) was activated in response to cellular stresses in *Schizosaccharomyces pombe* [61]. We therefore hypothesize that, unlike the high-temperature group, mitochondrial function was not impaired in the HW group, and energy supply was largely normal to support the highly energy-consuming metabolic processes.

### 4.2. Shared Genes and KEGG Pathways

The *HSP* is the most well-known protein family produced in response to heat stress [34,62,63]. In our study, genes including *HSP75* and *HSPD1* were shown to be downregulated in these acclimated groups, but DnaJ heat shock protein family (*Hsp40*) homolog subfamily C member 12 (*DNAJC12*) were up in the H group. Meanwhile, heat shock 70 kDa protein (*HSPA1s*), heat shock protein 110 kDa (*HSP110*), and molecular chaperone HtpG (*HSP90A*) were not significantly changed. These are unexpected results. HSP genes were likewise downregulated in *E. tynerensis* (Oklahoma salamander) [35] and *B. otai* tadpoles [23], but upregulated in *Quasipaa spinosa* [64] under heat stress. Heat shock proteins are induced by a variety of stressors, despite their name referring to their reaction to heat stress. They appear to have protective properties and may aid in protecting cells against future problems. However, no clear explanation can be found for why they were downregulated in response to heat stress. The reduction in *HSP* expression in the liver shows that the protection provided by *HSP* induction after extreme temperature stress may be compromised. In addition, the duration of the stimulation affects the change in *HSP* expression [65]. For example, *E. tynerensis* mentioned above was treated for 30 days, whereas *B. otai* tadpoles were treated for 4 h, and *Q. spinosa* for time gradients of 0, 3, 6, 12, 24, and 48 h.

Ubiquitin modifies substrate proteins for destruction, which demands metabolic energy [66]. Low-expressed proteasome subunit genes may exist, including distemperedness of the ubiquitin system and lower proteasome in frogs. Endocrine disruptor-exposed *X. tropicalis* also had a decrease in liver proteasome activity [67] when faced with adverse circumstances.

Steroids are natural endogenous products that play the primary chemical messengers (hormones) role in regulating the metabolic, stress response, and reproductive functions [68]. Many genes were inefficient in steroid biosynthesis and steroid hormone biosynthesis. Triclosan (TCS) and triclocarban (TCC) disrupt steroid hormone action in frog and mammalian culture systems [69]. Steroid hormones foster communication during the frog breeding season [70]. Temperature and steroid hormones were explored on larval growth, development, and metamorphosis in *Bufo boreas,* suggesting that the effects of steroids depended on both age and temperature [71]. Even though steroids are vital, the suppressed steroid system implied that the drop in steroid or hormone-related genes was likely a reaction to the stress of high temperatures and heat waves in the liver of *N. pleskei*, which caused it to maintain an inactive organism state in order to survive.

## 5. Conclusions

Genes were more transcriptionally suppressed when exposed to high temperatures than heat waves, and the body stayed in low-energy states for combating long-term adverse environments to survive. It might be strategic to preserve initiation to executive protein activity under heat wave stress. Under both stress conditions, compromised protection of *HSP* and sluggish steroid activity occurred in frogs. *Nanorana pleskei* are affected more seriously by high temperatures than by heat waves; thus, a threat to frogs grows due to trends in global warming. The information gathered in this study may be used as a basic guide for assessing the possible adverse ecological impacts on frogs, which is beneficial for the long-term preservation or future predictions of amphibian species.

## Data Availability

The RNA-Seq data generated and analyzed in this study are available in the National Center for Biotechnology Information (NCBI) Sequence Read Archive (SRA), BioProject ID number PRJNA837749, BioSample accession numbers SAMN28231190-SAMN28231198.

## Supplementary Materials

The following supporting information can be downloaded at: https://www.mdpi.com/article/10.3390/cimb45040192/s1. Table S1: The specific information of each sample. Table S2: KEGG functional enrichment results of up- and down-regulated DEGs. Table S3: GO functional enrichment results of up- and down-regulated DEGs. Table S4: Detailed information on differentially expressed genes (DEGs).
